# Evaluation of prognostic factors in patients with Bell's palsy

**DOI:** 10.1002/brb3.2385

**Published:** 2021-09-27

**Authors:** Dilli Ram Kafle, Sanjeev Kumar Thakur

**Affiliations:** ^1^ Institute of Neuroscience Nobel Medical College Biratnagar Nepal; ^2^ Department of ENT Nobel Medical College Biratnagar Nepal

**Keywords:** Bell's palsy, House–Brackmann grade, nerve conduction study

## Abstract

**Background:**

Bell's palsy is a common neurological problem that leads to peripheral palsy of the facial nerve. Most patients have a favorable response with or without treatment while some are left with significant facial deformity. Identification of factors which influence the outcome in patients with Bell's palsy may help clinicians counsel better.

**Methods:**

A prospective cross‐sectional study was carried out in the Department of Neurology Nobel Medical College, Biratnagar, between February 2020 and February 2021 after obtaining ethical clearance from the institutional review committee. Patients were assessed at the time of presentation to hospital and followed up at 1 week, 1 month, and 3 months after the onset of illness to evaluate for recovery.

**Results:**

Sixty‐two (61.4%) of 101 patients had a favorable outcome at the follow up on the third month, achieving H‐B grade 2 or lower, while 33 (32.7%) had moderate dysfunction and 6 (5.9%) had severe dysfunction. The following factors were associated with favorable outcome: younger age of onset (*p* < .001), lower initial H‐B grade of III or IV (*p* = .001), lesser degree of amplitude reduction on affected side as compared to unaffected side (*p* = .001) and absence of hypertension and diabetes. The following factors did not influence outcome at three months: duration of Bell's palsy (*p* = 0.142), side of face affected, and gender (*p* = .09).

**Conclusions:**

Most of the patients with Bell's palsy have favorable outcomes. Age, hypertension, initial H‐B grade, and extent of facial nerve degeneration as recorded by nerve conduction studies are important predictors of outcome.

## Abstract 2

Most of the patients with Bell's palsy have favorable outcomes. Age, hypertension, initial H‐B grade, and extent of facial nerve degeneration as recorded by nerve conduction studies are important predictors of outcome.

## 1 INTRODUCTION

Bell's palsy is the most common cause of acute mono‐neuropathy (15), with an annual incidence of 20 to 30 per 100,000 individuals (1; 19). It can occur at any age, with an average age of onset of 40 years (9). Facial weakness is usually unilateral (23). There is no difference in the side of the face affected nor is there a seasonal predominance (8; 9). Up to 30% of the patients with Bell's palsy develop long‐term disability (15; 14).

The outcome of Bell's palsy depends on the severity of facial nerve degeneration which is best seen using nerve conduction studies (NCS) (18). Reductions in the amplitude of compound motor action potential (CMAP) of the affected nerve compared to the unaffected side denotes the severity of facial nerve involvement (13; 7).

We conducted this study to evaluate if facial nerve conduction study findings, treatment modality, gender, age of onset, hypertension, and diabetes mellitus influenced the outcome.

## 2 METHOD

This was a prospective cross‐sectional study carried out at the Department of Neurology, Nobel Medical College, Biratnagar, Nepal between February 2020 and February 2021.

All the patients with Bell's palsy who were presented to the hospital were included in this study.

Patients in whom the cause of facial paralysis (e.g., neoplasm, trauma, otitis media, Ramsay‐Hunt syndrome) was known were excluded from the study.

All the patients included in this study had a total of four visits. The first visit was when the patient was first seen by us. House‐Brackmann facial nerve grading scale has been commonly used to assess the severity of facial weakness (6), where grade 1 indicates normal power while grade 6 indicates complete paralysis of the facial nerve. Nerve conduction study was done on day 4 or 5 after the onset of facial paralysis. As nerve conduction study can be affected by room temperature, a constant temperature of the examination room was maintained with the use of an air conditioner. The amplitude reduction of the affected side was compared with the unaffected side and was expressed as percentage reduction. A poor nerve conduction study result was defined as a loss of amplitude greater than 90%, whereas loss of 90% or less was classified as a good outcome (21).

Patients were treated with either prednisolone alone, or a combination of acyclovir 400 mg five times a day and prednisolone 1 mg/kg per day for 5 days and were gradually tapered over the next 5 days. Facial physiotherapy was offered to all patients irrespective of severity of facial weakness.

The second visit was 1 week after the first visit. The third visit at 1 month and the last visit was at 3 months after the onset of facial weakness. Baseline characteristics of the patients that were assessed during each visit included severity of facial paralysis before initiating treatment and at 3 month follow up. Demographic factors including age, sex, and duration of onset of facial paralysis, previous history of facial palsy, history of smoking, alcohol abuse, diabetes mellitus, and hypertension were also recorded. Hypertension was defined as blood pressure more than 140/90 mm Hg.

Ethical clearance for the study was obtained from the institutional review committee of Nobel Medical College.

The degree of facial paralysis at first visit was categorized as mild (H‐B grade II), moderate (H‐B grades III–IV), or severe (H‐B grade ≥ V). We assessed the H‐B grade at 3 month follow up in all patients and defined favorable outcome as an H‐B grade of I or II and unfavorable outcome as those with H‐B grade III or higher.

The statistical analysis which was used was the chi‐square test for nominal non‐parametric data. Pearson correlation was used for quantitative data. A *p*‐value of less than .05 was considered significant.

## 3 RESULTS

Majority of the study population was adult with a mean age of 42 ± 18. Almost equal number of patients had either left or right side of the face affected as shown in Table 1.

**TABLE 1:** Clinical characteristics of the patients

Clinical characteristics	Number of patients (*N* = 101)
Male	59 (58.2%)
Female	42 (41.58%)
Age	42 ± 18.9
Blood pressure during examination	
Normal	74 (73.3%)
High	27 (26.7%)
Side of face affected	
Right	51 (50.5%)
Left	50 (49.5%)
Duration of onset of facial palsy (days)	6 ± 4.24
History of diabetes mellitus	
Present	16 (15.8%)
Absent	85 (84.2%)
History of hypertension	
Present	18 (17.8%)
Absent	83 (82.2%)
Past history of Bell's palsy	12 (11.9%)
History of smoking	10 (9.9%)
History of alcohol consumption	12 (11.9%)

Most of the patients were adults mainly in the age range of 20 to 40. Patients less than 20 years and more than 80 years of age constituted 13 (12.86%) as shown in Table 2. Age was an important determinant of outcome, with advancing age having worse outcome than younger patients (*p* < .001).

**TABLE 2:** Age of the study population

Age	Number of patients (*N* = 101)
<20	9 (8.9%)
20–39	43 (42.6%)
40–59	29 (28.7%)
60–79	16 (15.8%)
≥80	4 (3.96%)

House‐Brackmann grading system was used to assess the severity of facial weakness as shown in Table 3. Lower H‐B grade at the time of presentation to hospital was associated with favorable outcome while higher H‐B grade was associated with worse prognosis (*p *< .001).

**TABLE 3:** H‐B grade

H‐B grade	At onset	At 3 month follow up
≤2	8 (7.9%)	62 (61.4%)
3 and 4	51 (50.5%)	33 (32.7%)
≥5	42 (41.6%)	6 (5.9%)

Nerve conduction study was done in all the patients with Bell's palsy which showed reduction in the amplitude of the compound muscle action potential as shown in Table 4. We found that a greater degree of amplitude reduction was associated with worse outcome (*p* = .001).

**TABLE 4:** Amplitude reduction of compound muscle action potential

Amplitude reduction	Number of patients
<20	22 (21.8%)
20–39	21 (20.8%)
40–59	29 (28.7%)
60–79	16 (15.8%)
≥80	13 (12.9%)

**TABLE 5:** Treatment offered to the patients

Treatment	Number of patients
Prednisolone and physiotherapy	44 (43.6%)
Prednisolone plus acyclovir and physiotherapy	57 (56.4%)

There was no statistically significant difference in recovery of facial function between patients who were treated with steroid combined with acyclovir and facial physical therapy, and those who were treated with steroid combined with physiotherapy (*p* = .07).

## 4 DISCUSSION

We assessed several factors including age, sex, duration of facial palsy, initial H‐B grade, comorbid conditions such as hypertension and diabetes, nerve conduction study findings, and treatment offered to the patients. Several studies were done in the past to evaluate factors affecting outcome in patients with Bell's palsy.

The exact cause of Bell's palsy remains known. During decompressive surgery for Bell's palsy, edema of the facial nerve palsy has been observed (4), which is consistent with finding of MRI enhancement of the facial nerve (24). Although Bell's palsy is idiopathic by definition, increasing evidence of a viral etiology, namely the herpes simplex virus, has been found (16).

In our study, we did not find any effect of sex on the outcome (*p* = .091). However, age was an important determinant of outcome, with advancing age having worse outcome than younger patients (*p* < .001).

Smith and Cull (20) evaluated the association of healing process with regeneration and central adaptation which has been found to decrease with advancing age. This explains the poor prognosis in elder patients. Peitersen (15) reported that a patient's age at the time of complete or incomplete paralysis was associated with treatment outcome for Bell's palsy.

We assessed blood pressure in all the patients at the time of initial examination and during each visit to the hospital, and determined if it affected the outcome. We found that hypertension at the time of onset of Bell's palsy measured during initial presentation to hospital was significantly associated with worse outcome (*p* = .03). In three adult case studies in patients with known hypertension, facial palsy was found to occur during episodes of raised blood pressure due to non‐adherence to medicine (2; 5; 10). Bell's palsy resolved when the blood pressure was brought under control, suggesting an association between controlled blood pressure and recovery of Bell's palsy. Lee et al. (11) had shown that initial severity of Bell's palsy and control of blood pressure were factors associated with good outcome. When hypertension was detected, medications to control blood pressure was initiated as indicated.

In patients with Bell palsy, diabetes was found to be associated with a poor outcome (*p* = .007). In patients with diabetes mellitus, Takemoto et al. (22) found worse outcomes than those who did not have diabetes. Chronic hyperglycemia in patients with diabetes mellitus affects the facial nerve fibers with negative effects on outcome in patients with Bell's palsy.

In our study, both left and right sides were almost equally affected. However, the side of the face affected did not alter the ultimate outcome in patients with Bell's palsy. Similarly, duration of Bell's palsy before presentation to hospital also did not have a significant effect on the outcome. Patients had a longer duration of illness before coming to the hospital because Nepal is a mountainous country with difficulty in traveling from remote areas.

Twelve (11.9%) patients had previous history of facial palsy, either on the same or opposite side. This figure is higher than that mentioned in other studies.

We used the H‐B grading system in our study as it is the most frequently used system to evaluate the degree of facial function in Bell's palsy. We used H‐B grade 2 or lower as having favorable outcome in terms of facial function. Lower H‐B grade was associated with favorable outcome while higher H‐B grade was associated with worse prognosis (*p* < .001). A similar finding was reported by Yoo et al. (25), with lower H‐B grade resulting in better outcome.

Nerve conduction study test has been used to evaluate the severity of facial nerve damage that quantifies the facial nerve function indirectly by recording motor unit action potential (MUAP) and compound muscle action potentials (CMAP) (12). By comparing the maximum amplitude of the compound muscle action potential of the affected side with the amplitude from the unaffected side, the amount of the degenerated nerve can be assessed. We found that greater degree of amplitude reduction was associated with worse outcome (*p* = .001). A study done by Prakash and Raymond (17) found that about 65% of those with <50% facial nerve degeneration had complete clinical recovery within a month and more than 90% of those with <75% degeneration had complete recovery within 2 months.

In our study, there was no statistically significant difference in recovery of facial function between patients who were treated with steroid combined with acyclovir and facial physical therapy and those who were treated with steroid combined with physiotherapy (*p* = .07). According to the American Academy of Otolaryngology guideline, treatment of patients with Bell's palsy with oral corticosteroid within 3 days of facial weakness is likely to be very effective in patients with or without the use of concurrent antiviral therapy (3).

## 5 CONCLUSIONS

Bell's palsy is a common neurological problem which causes a lot of anxiety to patients. A good history and examination help to differentiate peripheral cause of facial palsy from central cause. Age, diabetes mellitus, hypertension, initial H‐B grade, and extent of facial nerve degeneration as recorded by nerve conduction studies, affect clinical outcome of Bell's palsy at 3 months after its onset. Our study helps to identify specific factors in the patients which may influence outcome.

### PEER REVIEW

The peer review history for this article is available at https://publons.com/publon/10.1002/brb3.2385.

## Data Availability

Data supporting the findings of this study are available upon reasonable request to the author.
